# Non-Destructive Quality Assessment of Tomato Paste by Using Portable Mid-Infrared Spectroscopy and Multivariate Analysis

**DOI:** 10.3390/foods9091300

**Published:** 2020-09-15

**Authors:** Didem Peren Aykas, Karla Rodrigues Borba, Luis E. Rodriguez-Saona

**Affiliations:** 1 Department of Food Science and Technology, The Ohio State University, 100 Parker Food Science and Technology Building, 2015 Fyffe Road, Columbus, OH 43210, USA; aykas.1@osu.edu; 2 Department of Food Engineering, Faculty of Engineering, Adnan Menderes University, Aydin 09100, Turkey; 3 Department of Food and Nutrition, São Paulo State University, Araraquara 01049-10, Brazil; borbakr@gmail.com

**Keywords:** tomato paste, quality traits, natural tomato soluble solids, Bostwick consistency serum viscosity, lycopene, FT-IR, PLSR

## Abstract

This research aims to provide simultaneous predictions of tomato paste’s multiple quality traits without any sample preparation by using a field-deployable portable infrared spectrometer. A total of 1843 tomato paste samples were supplied by four different leading tomato processors in California, USA, over the tomato seasons of 2015, 2016, 2017, and 2019. The reference levels of quality traits including, natural tomato soluble solids (NTSS), pH, Bostwick consistency, titratable acidity (TA), serum viscosity, lycopene, glucose, fructose, ascorbic acid, and citric acid were determined by official methods. A portable FT-IR spectrometer with a triple-reflection diamond ATR sampling system was used to directly collect mid-infrared spectra. The calibration and external validation models were developed by using partial least square regression (PLSR). The evaluation of models was conducted on a randomly selected external validation set. A high correlation (R_CV_ = 0.85–0.99) between the reference values and FT-IR predicted values was observed from PLSR models. The standard errors of prediction were low (SEP = 0.04–35.11), and good predictive performances (RPD = 1.8–7.3) were achieved. Proposed FT-IR technology can be ideal for routine in-plant assessment of the tomato paste quality that would provide the tomato processors with accurate results in shorter time and lower cost.

## 1. Introduction

California produces 96% of the total USA processed tomato products (11–15 million tons annually), and that number represents 30% of the world’s total production [1]. Processed tomatoes are used to make tomato products such as juice, ketchup, sauces, but they are mainly (75%) concentrated into a paste [2,3]. The concentrated paste is generally stored up to 2 years and sold as it is or diluted into value-added products, including sauces, salsas, or ketchup [3,4,5].

Tomato paste is the main constituent in tomato products, and thus, monitoring and retaining the quality traits during the production process is very important [5,6]. Routine quality control practices involve hourly testing of freshly manufactured tomato paste samples from each production line. These tests include soluble solids, viscosity, consistency, pH, acidity, and color [5,7]. Bostwick consistency or gross viscosity and serum viscosity are the pre-eminent quality parameters of tomato paste in determining consumer’s acceptability and are an essential part of the quality grade standards [8]. Besides having a crucial part in the end-product quality and acceptability, consistency and viscosity also have crucial economic implications for the tomato industry because processed tomatoes with higher consistency and viscosity lower the production costs by decreasing the amount of tomato needed to produce a certain level of quality product [8].

The natural tomato soluble solids (NTSS) in fresh tomatoes is mainly contributed by the reducing sugars, which significantly affects the overall quality of the final product, as well as its yield and consistency [8,9]. The solid content in tomato paste is mainly affected by the degree of concentration, and to some extent, by the cultivar [10]. Similar to the Bostwick consistency and serum viscosity, fresh tomatoes with a higher NTSS content require less tomato fruit and less water removal to reach the desired end-product quality [8,11]. Sugars and organic acids in tomatoes and their interactions with the volatile compounds are responsible for the typical sweet-sour flavor of tomatoes or tomato products [12,13]. Glucose and fructose are the main sugars presented in tomato paste with a small amount of raffinose, arabinose, xylose, and galactose [8]. Two other important quality parameters in tomato paste are pH and titratable acidity (TA), which play a key role in food safety and the tomato flavor. Tomato paste’s pH and the acidity are affected by the cultivar, the ripening stage at the time of harvesting, the tomato processing conditions (hot vs. cold break processing), growing location, and seasonal variations [2,8,9,14]. Tomatoes are low acidic foods (pH < 4.6), and they do not require extreme thermal treatment to ensure microbiological food safety; furthermore, the low pH helps to inhibit the spore-forming bacteria *Bacillus coagulans*, making it less likely that the “flat sour” spoilage is formed [2,10]. A pH of 4.4 is considered the highest desirable value for food safety and to prevent thermophilic spoilage [15,16,17]. The industrial tomato processors in California targets a pH 4.2–4.3 range to ensure a margin of safety [2]. Citric acid is the main acid in tomatoes and contributes to the pH and the titratable acidity [11]. Fresh tomatoes are good sources of vitamin C, but are highly susceptible towards thermal degradation, therefore during the paste production process, tomatoes lose about half of their vitamin C content because of the heat applications and processing [18,19].

In consideration of the role played by solids, viscosity, pH, sugars, and acids in tomato quality, several analytical methods have been developed and are currently used in the tomato industry. However, these traditional techniques are time-consuming, require laborious sample preparation, use of hazardous solvents, and high skilled testing personnel, also their applications are limited to the laboratory environments, and they are not well adapted to routine in-line quality analysis [11]. Each test provides only one quality trait information at a time, and currently there are still no truly standardized protocols for the routine determination of quality parameters in the tomato industry, and there is a need for standardized protocols. Thus, the development of rapid, cost-effective, and robust techniques is required for the quality control of tomato products.

Infrared spectroscopy is a promising technique for accurately assessing chemical profiling of raw materials and final products in the agro-food sector with simplicity, time- and cost-efficiency [11]. The mid-infrared (4000–400 cm^−1^) region provides powerful vibrational information regarding the functional groups in different components and resulted in determining chemical compounds of interest in the food matrix. Advancements in miniaturization techniques, adaptable sampling accessories including attenuated total reflectance (ATR), or transmission cell (dial-path) with the corporation of chemometric techniques offer a valuable tool to tomato processors and breeders for the rapid assessment of tomato quality traits with real-time screening and field-based assessments. Near-Infrared (NIR) spectroscopy has been applied for the quantitative determination of soluble solids, pH, sugars, TA, organic acids, firmness, lycopene, and β-carotene in fresh tomatoes [20,21,22,23,24,25,26] and in dehydrated tomatoes [27]. Similarly, FT-IR analysis has been successfully implemented to estimate the compositional parameters in fresh tomatoes [9,11,13,28,29,30]. However, none of the previous studies evaluated the application of portable FT-IR spectroscopy on quality parameters of tomato paste, which could be invaluable for tomato paste processors. It is essential to include a vast array of samples rich in compositional variation through the collection from different processors and years to build a robust calibration model [31].

This study aimed to evaluate the use of the field-deployable portable infrared sensor to the simultaneous prediction of multiple quality traits of tomato paste (natural tomato soluble solids, pH, Bostwick consistency, titratable acidity, serum viscosity, lycopene, glucose, fructose, ascorbic, and citric acid) without any sample preparation.

## 2. Materials and Methods

### 2.1. Tomato Paste Samples

A total of 1843 tomato paste samples were provided by four different major tomato processors in California, USA, for 2015, 2016, 2017, and 2019. Tomato paste samples were thermally processed, and there was no addition of any food additive.

### 2.2. Reference Analyses

The reference values for natural tomato soluble solids (NTSS), pH, Bostwick consistency, titratable acidity (TA), and serum viscosity were provided by the quality assurance department of each tomato processor company. In contrast, the quantification of lycopene, individual sugars (glucose and fructose), and acids (ascorbic and citric) was done at the Food Science and Technology department laboratories at The Ohio State University (Columbus, OH, USA). Freshly produced tomato paste samples were sent by overnight carrier in insulated boxes with dry ice to sustain the refrigerated temperatures. The NTSS, pH, Bostwick consistency, TA, and serum viscosity tests were performed within the next hour of the paste production. On the other hand, overnight shipped samples were stored in refrigerated conditions (4 °C) until analyzed for lycopene, sugars, and acid, that were done within a week of receiving the shipments.

The NTSS measurements were carried out by filling (¾ full) microcentrifuge tubes with non-diluted tomato paste samples. The microcentrifuge tubes were rotated using a Sorvall-Discovery M150 SE microcentrifuge (Thermo Fisher Scientific, Waltham, MA, USA) at 140,000 rpm for 10 min. Once the run is over, the serum portion (upper part) of the sample from each tube was slowly pipetted onto the prism of the temperature-controlled refractometer (RX 5000i ATAGO, Bellevue, WA, USA) and the measurement at 25 °C were recorded. The pH of tomato paste samples was determined using a Metrohm 827 pH meter (Herisau, Switzerland) at 25 °C. Samples were diluted with deionized water until reaching 12 °Brix in a glass beaker. Bostwick consistency or gross viscosity of tomato paste samples were determined using a Bostwick consistometer. Paste samples were diluted with deionized water until reaching 12 °Brix and approximately getting 200 g of final solution weight. The mixture was placed into a stomacher blender (Seward™ Stomacher™ Model 400, West Sussex, UK) to obtain a completely homogenous blend. The mixture was cooled or heated to 20 °C (68 °F), and the chamber of the Bostwick consistometer was filled with the mixture. The diluted paste placed in the chamber was leveled by removing the excessive sample with a spatula. The gate of the consistometer was released, and the sample flow after 30 s was recorded to the closest 0.1 cm sign. Titratable acidity (TA) were determined with an Easy pH automatic titrator (Mettler Toledo, Columbus, OH, USA) by mixing 10 g of tomato paste with 100 mL deionized water and titrating with 0.1 N NaOH. TA results were reported as g citric acid/100 g sample or in %. The samples’ serum viscosity was determined by bringing the paste samples to 6 °Brix and centrifuging them at 2000 rpm for 15 min Thermo Fisher Scientific, Waltham, MA, USA). The supernatant portion of the sample was filtered through a Whatman no.1 filter paper (Whatman PLC, Maidstone, UK), and the filtrate carefully poured into the large orifice of the Cannon-Fenske viscometer until the line marked in the reservoir. The viscometer was placed into the 30 °C ± 1 °C water bath for 5 min for temperature equilibration. The time in seconds it takes for the meniscus to move from the graduated mark above the lower bulb to the graduated mark below the lower bulb was recorded, while both orifices open to the air. The serum viscosity was reported as centistokes (cSt) and calculated using distilled water’s absolute viscosity under the same conditions. All analyses were conducted in duplicate.

Lycopene concentrations in tomato paste samples were determined following the procedure described by Anthon and Barrett (2007) with some changes [32]. A total of 0.3 g of tomato paste sample was diluted with 1 mL of deionized water and vortexed for 1 min. A total of 0.1 g of diluted tomato paste was transferred to another centrifuge tube and 8 mL of hexane/ethanol/acetone (HEA) 2:1:1 (*v*/*v*/*v*) solvent mixture was added and vortexed for 45 s. The mixture was kept for 20 min in the dark to avoid lycopene degradation through light irradiation [33]. One mL of deionized water was added and vortexed for 15 s and stored for 10 min in the dark for phase separation and let the air bubbles disappear. Absorbance readings were performed using Cary 50 UV-Vis spectrophotometer (Agilent Technologies Inc., Santa Clara, CA, USA). The equipment was set to 503 nm, which is the maximum absorption wavelength for lycopene. The equipment was set to zero using a blank prepared with water (instead of tomato paste) and HEA solvent mixture. Lycopene analysis for the extracted samples was conducted in duplicate. The concentration of the lycopene was calculated using the extinction coefficient (172 mM^−1^) and absorbance readings.

Individual sugars, including glucose and fructose concentrations, were simultaneously determined using high-performance liquid chromatography (HPLC) (Shimadzu, Columbia, MD, USA) equipped with a CBM-20A controller, an LC-6AD pump, a SIL-20AHT autosampler, a CTO-20A oven, and a RID-10A refractive index detector. Paste samples (0.2 g) were weighed into a micro-centrifuge tube and diluted with HPLC grade water (1.6 mL). The mixture was vortexed for 30 s and centrifuged at 13,200 rpm for 15 min at 25 °C. The supernatant part was filtered through a 0.45 μm pore size filter (Phenomenex^®^, Torrance, CA, USA) into a glass amber HPLC vial. Extracted sugars were separated through a Rezex RCM-Monosaccharide Ca^+^ 300 × 7.8 mm column (Phenomenex^®^, Torrance, CA, USA). The elution of the sugars was carried out isocratically using HPLC grade water as a mobile phase at a flow rate of 1 mL/min for 20 min at 80 °C. Chromatograms were automatically integrated using LC Solutions software (Version 3.0, Shimadzu, Columbia, MD, USA). A standard curve was generated to calculate the individual sugars, with a concentration range from 1.56 to 50 mg/mL (>99% purity, Fisher Scientific, Fair Lawn, NJ, USA). Sugar analysis was performed in duplicate.

Organic acids, including ascorbic and citric acid concentrations, were determined by an HPLC (1100 Series, Agilent Technologies, Santa Clara, CA, USA) composed of a G1311A quaternary pump, a G1322A degasser, a G1313 ALS autosampler, a G1316A column compartment, and a G1315B diode array detector. Samples were prepared to mix tomato paste (0.3 g) with 4.5% metaphosphoric acid (1.5 mL) (Fisher Scientific, Fair Lawn, NJ, USA) into a 2 mL microcentrifuge tube and vortexed for 30 s. A total of 100 μL of 100 Mm tris (2-carboxyethyl) phosphine (TCEP) (Sigma Aldrich, St. Louis, MO, USA) was added into the centrifuge tube in order to reduce dehydroascorbic acid to ascorbic acid and expand the stability of ascorbic acid [34], samples were incubated at 4 °C for 8 h and centrifuged at 10,000 rpm for 15 min at 4 °C. Similar to the sugar analysis, supernatant filtered through a 0.45 μm pore size filter (Phenomenex^®^, Torrance, CA, USA) into a glass amber HPLC vial. The elution of the acids was succeeded through a Prevail™ 5 µ, 150 × 4.6 mm column (Hichrom, Berkshire, UK). A total of 10 µL sample was injected through the column; pH 2.2 adjusted HPLC grade water was used as a mobile phase with a consistent flow rate of 0.8 mL/min. Chromatograms were automatically integrated for citric acid at 210 nm and ascorbic acid at 245 nm using ChemStation software (Agilent Technologies, Santa Clara, CA, USA). A standard curve was generated to calculate individual organic acids (Sigma Aldrich, St. Louis, MO, USA). The acid analysis was performed in duplicate.

### 2.3. Mid-Infrared Analysis

The spectral collection was carried out in California at each tomato processor’s quality assurance laboratory, right after the paste production, to minimize any change in the reference values. The mid-infrared analysis was carried out using a portable FT-IR sensor (Agilent Technologies, Santa Clara, CA, USA) coupled with triple-reflection diamond Attenuated Total Reflectance (ATR) crystal. The ATR has a 2 mm diameter sampling surface with a 200 µm active area, which offers ~6 µm effective depth of penetration for IR energy at 1700 cm^−1^. The FT-IR unit is also equipped with a Zinc Selenide (ZnSe) beam splitter and a thermo-electrically cooled deuterated triglycine sulfate (DTGS) detector. Spectra were collected from 4000 to 650 cm^−1^ with a resolution of 4 cm^−1^. To increase the signal-to-noise ratio, 64 spectra were co-added in each sample collection, and between every measurement, a spectral background was taken to eliminate the environmental changes. Approximately 0.5 gr of tomato paste sample was directly applied to the active area of the sampling surface, ensuring full coverage of the sample is achieved. The spectral collection was done in duplication for each sample, and collected spectral data were recorded by using Agilent MicroLab PC software (Agilent Technologies, Danbury, CT, USA).

### 2.4. Partial Least Squares Regression (PLSR) Analysis

The spectral data were imported as GRAMS (.spc) files from the FT-IR instrument and evaluated using Pirouette^®^ comprehensive chemometrics modeling software (version 4.5, Infometrix Inc., Bothell, WA, USA). Partial least squares regression (PLSR) with full cross-validation (leave-one-out approach) was employed to generate multivariate quantitative models to quantify the concentrations of quality traits in tomato paste using a portable FT-IR unit. The spectral data were transformed by the mean-center, smoothing (35-points), and Savitsky–Golay second derivative (35-points). PLSR establishes linear correlations between the spectral data and the reference values, which maximizes their covariance [35]. PLSR sifts the most useful information from a large number of spectral data points into the first several partial least square (PLS) factors (or latent variables—LVs), where the background effects can be present in the less important factors [36]. The latent variables are the orthogonal factors, which provide the highest correlation with the dependent variable. The performance of the regression models was evaluated by the number of LVs, scores, loadings, standard error of cross-validation (SECV), the coefficient of determination (R-value), standard error of prediction (SEP), and outlier diagnostics. In contrast, outliers were determined using residual and Mahalanobis distances. The accuracy of the calibration models increases with the increasing number of the LVs or factors in the model at first, but then decreases as a result of overfitting the data by adding noise, which makes the models ineffective [37]. On the other hand, selecting too few LVs yields an under-fitted model that integrates insufficient information of the data. Therefore, the optimal number of LVs-factors should be chosen through the cross-validation approach by plotting the SECV against the PLSR factors.

## 3. Results and Discussion

### 3.1. Reference Values in Tomato Paste Samples

The reference compositional values for ten main quality traits of tomato paste are summarized in Table 1. The inclusion of a large number of paste samples from different years and different processing plants gave a wide range of reference values (Table 1). We should also point out that measurements of the quality parameters were performed at different laboratories using different equipment operated by various individuals, which may introduce variation in the reported results. In general, the values that were obtained in this study were similar to those reported in the literature. Overall the values ranged from 24.1–38.1 (29.4 ± 3.0) °Brix (natural tomato soluble solids—NTSS), 4.14–4.49 (4.37 ± 0.07) for pH, 0.8–11.9 (3.6 ± 1.9) cm for Bostwick consistency, 1.0–2.4 (1.6 ± 0.2) % citric acid for titratable acidity—TA, 64.6–977.2 (317.3 ± 192.8) cSt for serum viscosity, 400.6–869.1 (662.2 ± 94.7) mg/kg for lycopene, 67.5–128.2 (92.0 ± 11.9) g/L for glucose, 74.7–130.5 (94.4 ± 11.2) g/L for fructose, 12.1–110.7 (63.6 ± 20.0) mg/100 g for ascorbic acid, and 5.9–11.7 (8.2 ± 0.9) g/100 g for citric acid. Similar levels for tomato paste were also reported by other researchers [5,18,19,38,39,40,41,42]. According to the U.S. Department of Agriculture (USDA), the NTSS of the tomato paste should not be less than 24.0 °Brix (the maximum amount was not stated), and all the tested tomato paste samples were above that limit. Even though the minimum NTSS limit is 24.0 °Brix, the tomato paste producers in California adjust their productions per customer requirements. All producers except one (Company D) had NTSS at around 30 °Brix for all 4 years, but company D had an average NTSS of 26 °Brix. The pH of the tomato paste was low enough (pH < 4.6) to prevent any problem with pathogenic microorganisms. The pH of good quality pastes normally lies within the range of 4.0–4.4 to prevent problems with thermophilic microorganisms [8]. In general, paste producers in California target a range of 4.20–4.30 [2]. The average pH value from four different producers and years was 4.37 (Table 1). The range of Bostwick consistency in cm was larger (0.8–11.9) than those reported by Barrett and others (1998) who reported tomato paste from five different California county locations (3.1–5.5) [7]. Company A showed a larger variation in Bostwick consistency between years compared with other companies; on the other hand, all the companies’ Bostwick consistency averages showed similarities, except Company D (Table 1). Company D’s Bostwick consistency did not have large variation over the four years, but its consistency values were lower than that of the other companies (Table 1).

Titratable acidity (TA) of the samples did not show a big variance over the years or between different companies; only Company C showed slightly higher values for the average and the high end of the TA values (Table 1). The lycopene content in the tested samples ranged from 400.6 to 869.1 mg/kg of tomato paste was similar to reported values of 327–682 mg/kg [41]. Glucose and fructose content in the analyzed tomato paste samples ranged from 67.5 to 128.2 g/L and 74.7 to 130.5 g/L, respectively, which were higher than the values (57.5 g/L for glucose and 58.5 g/L for fructose) reported in the USDA nutrient database [43]. The USDA database provides information on commercial canned tomato paste, which is a reconstituted paste that is available on the market, made from concentrated tomato paste. However, the tomato paste samples used in this research were highly concentrated (up to 38.1 °Brix) and without any reformulation they may end up with higher values of individual and total reducing sugars. Ratios of fructose to glucose ranged from 1.0 to 1.1 and were similar to the ratio reported by others [8,44,45]. Companies did not show a large variation between years in terms of individual sugars, but Company D showed a lower sugar content than the other producers (Table 1).

In our study, vitamin C concentration varied over a large span within the same year; also, company D showed the lowest vitamin C values for all four years (Table 1). vitamin C content in tomato paste samples ranged between 12.1–110.7 mg/100 g, with an average of 63.6 ± 20.0 mg/100 g, which was about three times higher than reported in the USDA (2019) database (21.9 mg/100 g) [43]. USDA data did not provide any information about the studies referred to in their database, and vitamin C decreases with prolonged storage [3]. Tomato paste samples used in our study were analyzed immediately after the production. Our finding was comparable to ascorbic acid (67.5 mg/100 g) in tomato paste produced from California tomatoes [3]. Underwood (1950) employed a titration approach to determine vitamin C concentration in California tomatoes reporting ranges from 44 to 83 mg/100 g [46].

### 3.2. Spectral Information of Tomato Paste Samples.

**Figure 1:**
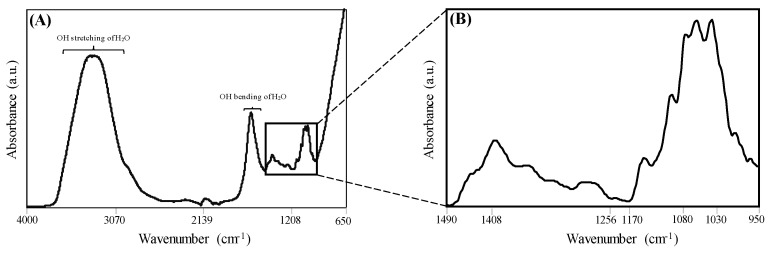
(**A**) A representative raw infrared absorption spectrum of tomato paste samples in the region of 4000–650 cm^−1^ and (**B**) the tomato paste spectrum in the 1490–950 cm^−1^ region utilized in chemometric analysis.

A typical FT-IR absorption spectrum collected in the mid-IR region (4000–650 cm^−1^) of a tomato paste sample is given in Figure 1A. The tomato paste samples’ spectral profile was very similar regardless of company or years; therefore, a single spectrum was given to exemplify the sample characteristics. The two broad bands seen within 3600–3000 cm^−1^ and 1700–1500 cm^−1^ were attributed to the stretching and bending of OH bonds in water, respectively [47]. The strong absorption bands centered at 1030, 1060, 1080, 1100, 1150, 1230, 1260, 1300, 1350, and 1408 cm^−1^ were shown in Figure 1B. The spectral region from 900 to 1150 cm^−1^ is mainly influenced by C-C and C-O stretching and C–O–H, C–O–C deformation of sugar, especially glucose and fructose in tomato paste [47,48,49,50]. The bands at 1030, 1060, and 1080 cm^−1^ associated with typical bending vibration of C-4-OH, C-1-OH, and C-1-H vibration of sugars, respectively [51,52]. The band at 1100 cm^−1^ attributed to the ν (C-O) in C-O-C group vibration [53] and the shoulder centered at 1150 cm^−1^ characteristic to a ring structure of a pyranose sugar, which is β-D-glucose in tomato paste [52,54]. The region from 1200 to 1474 cm^−1^ represents the bending vibrations of O-C-H, C-C-H, and C-O-H [50,55,56].

### 3.3. PLSR Calibration Models

The quantitative prediction models with PLSR based on the reference values for ten quality parameters were generated using infrared spectra from the portable FT-IR unit. Two subsets of data were created through randomized selection and assigned as calibration (80% of the total sample size) and external validation (remaining 20%) to evaluate the robustness of the created models. The statistical performance of each model used in the calibration and validation sets are presented in Table 2. The samples with high leverage and/or studentized residuals were identified as outliers and excluded from the model, resulting in the differences in the total number of samples for different quality attributes models. To improve each generated calibration model’s predictive capability, we removed frequency regions with low regression coefficients values since they are dominated by noisy and unreliable variables [13]. Furthermore, mathematical pre-processing procedures were employed to the spectra with the best performances obtained using mean-centering, Savitzki–Golay second derivative (35-window) and smoothing (35-window) transformations.

The optimal number of LVs or factors that give the lowest standard error of cross-validation (SECV) ranged between three to six (Table 2), explaining between 95 to 99% of the total variance. The correlation coefficient (R) quantifies the strength of the relationship among the measured and the predicted values. In general, a model with a higher correlation coefficient of cross-validation (Rcv) and lower SECV represents a model with a better prediction accuracy [9]. The generated PLSR models gave high correlation coefficients (Rcv ≥ 0.93) (Table 2) except the prediction models generated for pH (Rcv = 0.85). Furthermore, low prediction errors (SECV) for estimating the NTSS (0.44 °Brix), pH (0.04), Bostwick consistency (0.55 cm), TA (0.08% citric), serum viscosity (0.08 log cSt), lycopene (35.75 mg/kg), glucose (3.16 g/L), fructose (3.11 g/L), ascorbic acid (6.99 mg/100 g), citric acid (0.27 g/100 g) (Table 2), were obtained.

**Figure 2:**
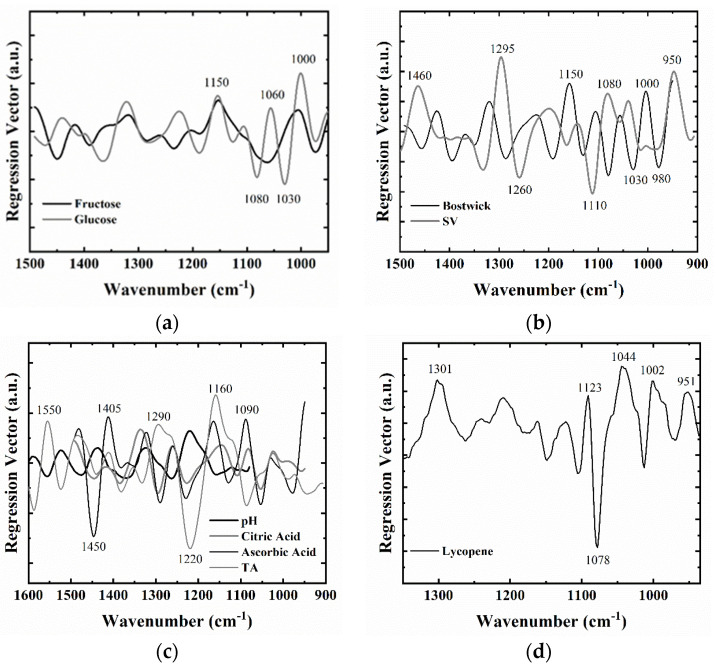
PLSR regression vectors for (**a**) glucose and fructose (**b**) Bostwick consistency and serum viscosity (**c**) titratable acidity, pH, citric acid, and ascorbic acid (**d**) lycopene.

The regression vector plots, presented in Figure 2, help differentiate functional groups with the highest variance responsible for the correlation between the reference levels and the IR spectra. The most important infrared regions for the tested quality parameters were in the 1500–950 cm^−1^ range with prominent peaks for titratable acids, citric and ascorbic acid observed at the 1450–1050 cm^−1^ region related with the absorption bands of C–O–H, C–C–H, and O–C–H bending modes of acids [28]. For the sugars in tomato paste, this region was dominated by COH group vibrations at 1080, 1060, and 1030 cm^−1^ [57].

Overall, our calibration models showed similar or superior performances with the studies in the literature that used FT-IR spectroscopy (portable dial-path or benchtop units) to predict soluble solids, pH, Bostwick consistency, TA, serum viscosity, lycopene, sugars, and acids in tomato juice [9,11,13,28,58] with fewer LVs.

**Figure 3:**
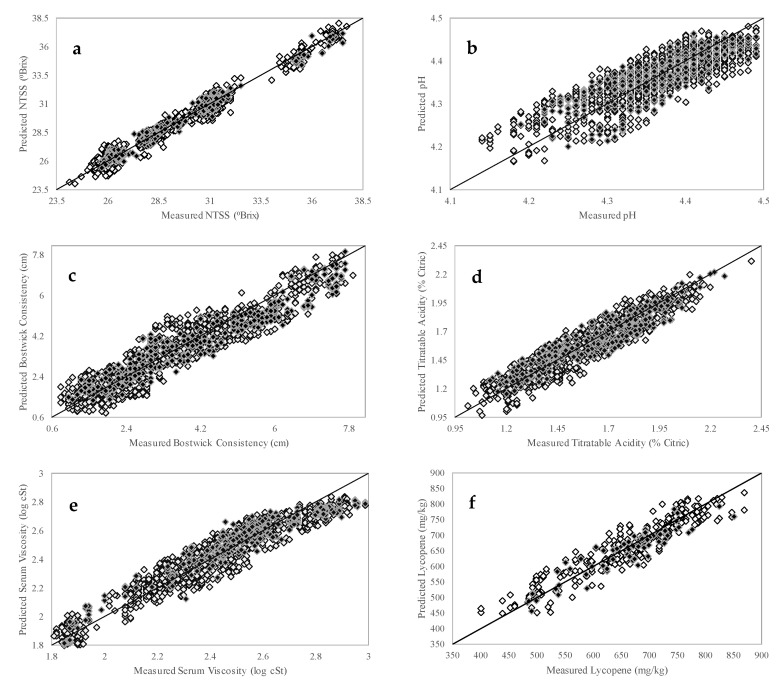

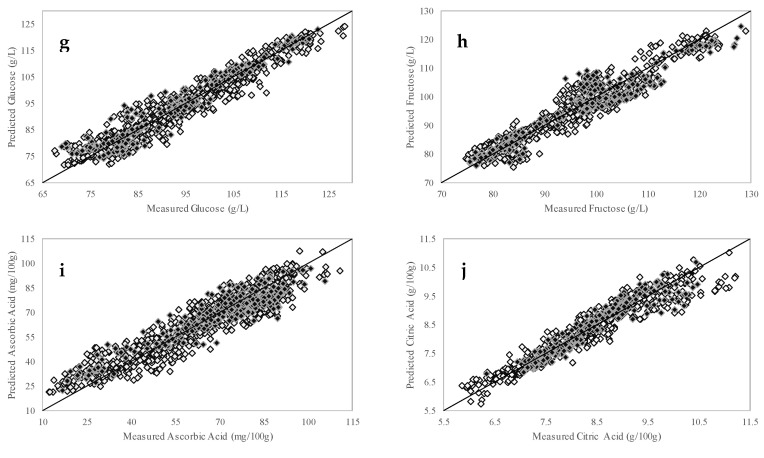
PLSR correlation plots for different quality traits in tomato paste using portable FT-IR unit equipped with a triple-reflectance ATR accessory (white and black diamonds represent calibration and validation set samples, respectively) (**a**) natural tomato soluble solids, (**b**) pH, (**c**) Bostwick consistency, (**d**) titratable acidity, (**e**) serum viscosity, (**f**) lycopene, (**g**) glucose, (**h**) fructose, (**i**) ascorbic acid, (**j**) citric acid.

The generated calibration models were validated by choosing a set of samples (20% of all the samples) that were not included in the original calibration population. This step called “external validation” was crucial to evaluate the robustness of the generated calibration model through an independent measure of equation accuracy expressed as the standard error of performance (SEP) [59]. After the evaluation of calibration and validation statistics, it was seen that similar SECV and SEP values were obtained through generated models (Table 2). Overall, for each quality trait, robust calibration models were generated with excellent predictive ability based on external validation. Additionally, Figure 3 demonstrates the correlation between the reference test results and FT-IR predicted levels of various quality traits. The white diamonds represent the independent calibration results; the black diamonds stand for the external validation set. Furthermore, to assess the models’ prediction performances, residual predictive deviation (RPD), which is the ratio between the standard deviation of the measured reference values to the SEP, was calculated. The RPD is a unitless value that provides information on how well the calibration model can predict new samples. The higher the RPD value the better the calibration model accuracy. The RPD values ranged between 1.8 to 7.3, with the highest RPD being obtained from the NTSS model.

## 4. Conclusions

The present study investigated the application of a portable and field-deployable FT-IR sensor in combination with pattern recognition analysis to predict multiple quality traits simultaneously without the need for any special sample preparation procedures. A total of 1843 samples were obtained from four different leading tomato paste processors in California, USA, from the processing years of 2015, 2016, 2017, and 2019. Generated calibration models were validated using an external validation set, and the robustness of the models was confirmed by obtaining similar SECV and SEP values. Our results were similar or superior to the studies that were conducted by using tomato juice. Proposed cutting-edge portable FT-IR technology offers the tomato industry a simple and high throughput technique that allows for the chemical profiling and prediction of physical characteristics of tomato paste all at the same time, which will help with production rate optimization and improve the quality, and safety of tomato products. Furthermore, the portable infrared spectrometers can become a valuable “out-of-the laboratory” analytical tool for food processors due to their robustness.

## Figures and Tables

**Table 1:** Reference method results of quality parameters in tomato paste samples from 4 different companies at the 2015, 2016, 2017, and 2019 production seasons.

Company	Year	Number of Samples		NTSS	pH	Bostwick	TA	Serum Viscosity ^c^	Lycopene	Glucose	Fructose	Ascorbic Acid	Citric Acid
A	2015	120	range	25.6–36.0	4.3–4.5	2.5–10.8	1.0–1.9	2.3–2.6					
			Avg ^a^ ± std ^b^	30.7 ± 2.8	4.4 ± 0.0	5.1 ± 2.0	1.5 ± 0.2	2.4 ± 0.1					
	2016	150	range	25.8–37.1	4.2–4.4	1.6–9.1	1.1–2.0	2.1–2.9		70.3–116.6	75.9–117.6	56.8–97.0	6.3–10.6
			avg ± std	30.1 ± 2.8	4.3 ± 0.1	4.1 ± 1.9	1.5 ± 0.2	2.6 ± 0.2		93.3 ± 10.3	96.4 ± 9.6	77.6 ± 8.9	8.4 ± 0.9
	2017	196	range	25.4–37.0	4.2–4.5	0.8–11.9	1.0–1.8	2.2–2.8		74.1–128.2	75.2–130.5	49.0–110.7	6.4–11.7
			avg ± std	28.6 ± 2.4	4.4 ± 0.0	3.5 ± 2.2	1.3 ± 0.2	2.5 ± 0.1		92.0 ± 11.5	92.3 ± 11.0	79.4 ± 10.9	8.4 ± 0.9
	2019	87	range	25.7–38.0	4.2–4.5	1.5–9.2	1.1–2.1	2.3–2.8	400.6–869.1				
			avg ± std	31.1 ± 2.8	4.4 ± 0.1	5.4 ± 2.1	1.4 ± 0.2	2.5 ± 0.2	644.5 ± 112.1				
	General	553	range	25.4–38.0	4.2–4.5	0.8–11.9	1.0–2.1	2.1–2.9		70.3–128.2	75.2–130.5	49.0–110.7	6.3–11.7
			avg ± std	29.8 ± 2.9	4.4 ± 0.1	4.3 ± 2.2	1.4 ± 0.2	2.5 ± 0.1		92.6 ± 11.0	94.1 ± 10.6	78.7 ± 10.1	8.4 ± 0.9
B	2016	79	range	28.5–37.5	4.1–4.5	4.0–7.6	1.3 -2.2	1.9–2.4		88.1–120.1	92.1–121.4	59.7–104.8	6.8–10.4
			avg ± std	32.2 ± 2.9	4.3 ± 0.1	5.6–1.2	1.6 ± 0.3	2.1 ± 0.2		104.0 ± 9.0	104.9 ± 7.8	75.6 ± 8.9	8.4 ± 0.9
	2017	116	range	28.0–36.2	4.1–4.5	1.1–6.4	1.3 -1.9	1.8–2.4		87.0–128.0	94.0–128.8	55.8–98.4	5.9–10.0
			avg ± std	30.6 ± 1.2	4.4 ± 0.1	4.8 ± 1.1	1.6 ± 0.1	2.1 ± 0.2		102.4 ± 6.0	101 ± 5.2	78.8 ± 11.1	8.3 ± 0.7
	2019	103	range	24.1–38.1	4.2–4.5	1.2–8.5	1.0–1.9	1.8–2.6					
			avg ± std	31.6 ± 4.3	4.4 ± 0.1	4.3 ± 2.1	1.5 ± 0.2	2.2 ± 0.2					
	General	298	range	24.1–38.1	4.1–4.5	1.1–8.5	1.0–2.2	1.8–2.6		87.0–128.0	92.1–128.8	55.8–104.8	5.9–10.4
			avg ± std	31.5 ± 3.0	4.3 ± 0.1	4.8 ± 1.6	1.6 ± 0.2	2.1 ± 0.2		102.0 ± 7.4	102.6 ± 6.6	77.5 ± 10.4	8.3 ± 0.8
C	2016	222	range	27.8–37.5	4.1–4.5	2.3–7.9	1.5–2.2	1.9–2.5		81.2–122.6	89.2–128.0	32.6–100.7	7.8–9.6
			avg ± std	31.0 ± 1.9	4.3 ± 0.1	4.4 ± 1.3	1.8 ± 0.2	2.2 ± 0.2		95.9 ± 8.8	101.0 ± 7.9	65.5 ± 12.8	8.6 ± 0.4
	2017	290	range	26.0–36.5	4.1–4.5	0.8–7.1	1.3–2.4	1.9–2.7		71.0–122.9	77.8–123.8	13.5–109.8	7.1–11.2
			avg ± std	29.1 ± 2.8	4.4 ± 0.1	3.1 ± 1.6	1.7 ± 0.2	2.4 ± 0.2		90.6 ± 12.6	94.4 ± 12.1	55.0 ± 19.9	8.5 ± 1.1
	2019	110	range	26.1–31.6	4.2–4.5	1.0–4.9	1.3–2.3	1.9–2.7	614.3–829.3				
			avg ± std	29.2 ± 2.1	4.4 ± 0.1	3.0 ± 1.2	1.7 ± 0.2	2.3 ± 0.2	690.9 ± 43.6				
	General	622	range	26.0–37.5	4.1–4.5	0.8–7.9	1.3–2.4	1.9–2.7		71.0–127.9	77.8–128.0	13.5–109.8	7.1–11.2
			avg ± std	29.8 ± 2.6	4.4 ± 0.1	3.5 ± 1.6	1.8 ± 0.2	2.3 ± 0.2		92.9 ± 11.4	97.3 ± 10.9	59.6 ± 17.9	8.6 ± 0.8
D	2015	47	range	25.0–26.6	4.2–4.4	1.1–2.4	1.3–1.5	2.6–2.7					
			avg ± std	25.9 ± 0.3	4.3 ± 0.0	1.8 ± 0.4	1.4 ± 0.0	2.7 ± 0.0					
	2016	48	range	25.3–26.5	4.4–4.5	2.3–2.9	1.3–1.4	2.9–3.0		76.9–84.6	78.1–89.0	43.9–63.4	5.9–7.1
			avg ± std	26 ± 0.3	4.4 ±0.0	2.6 ± 0.2	1.4 ± 0.0	3.0 ± 0.0		80.7 ± 1.8	83.5 ± 2.7	50.8 ± 5.3	6.5 ± 0.3
	2017	203	range	25.1–28.5	4.3–4.5	1.0–2.9	1.2–1.6	2.4–2.9		67.5–99.3	74.7–100.6	12.1–72.2	6.6–8.8
			avg ± std	26.2 ± 0.8	4.4 ± 0.0	1.9 ± 0.4	1.4 ± 0.1	2.7 ± 0.1		80.7 ± 6.9	82.4 ± 3.9	38.0 ± 13.0	7.4 ± 0.4
	2019	72	range	25.3–28.3	4.3–4.5	1.4–2.2	1.3–1.5	2.5–2.9					
			avg ± std	26.5 ± 1.0	4.4 ± 0.0	1.9 ± 0.2	1.4 ± 0.1	2.7 ± 0.1					
	General	370	range	25.0–28.5	4.2–4.5	1.0–2.9	1.2–1.6	2.4–3.0		67.5–104.3	74.7–100.6	12.1–72.2	5.9–8.8
			avg ± std	26.2 ± 0.8	4.4 ± 0.0	2.0 ± 0.5	1.4 ± 0.1	2.7 ± 0.1		80.7 ± 6.2	82.6 ± 3.7	40.4 ± 12.9	7.2 ± 0.5

^a^ mean. ^b^ standard deviation. ^c^ The unit of the serum viscosity results is log cSt. Lycopene analysis was only carried out in 2019 for two companies. Sugar and acid analyses with high-performance liquid chromatography (HPLC) were only carried out in 2016 and 2017. Each test was carried out in duplicate. NTSS: natural tomato soluble solids, TA: titratable acidity.

**Table 2:** Statistical performances of the partial least square regression (PLSR) models developed.

Parameter	Calibration Model					External Validation Model				
	Range	*N * ^a^	Factor	SECV ^b^	Rcv ^c^	Range	*N * ^d^	SEP ^e^	R_Pre_^f^	RPD ^g^
NTSS (°Brix)	24.1–38.1	1436	3	0.44	0.99	25.7–37.5	359	0.40	0.99	7.3
pH	4.14–4.49	1419	6	0.04	0.85	4.19–4.49	355	0.04	0.83	1.8
Bostwick Consistency (cm)	0.8–7.9	1382	5	0.55	0.94	1.0–7.7	345	0.58	0.96	2.9
Titratable Acidity (% Citric)	0.99–2.40	1406	6	0.08	0.94	1.12–2.27	352	0.09	0.93	2.8
Serum Viscosity (log cSt)	1.81–2.99	1304	6	0.08	0.96	1.85–2.99	326	0.08	0.96	3.5
Lycopene (mg/kg)	400.6–869.1	138	6	35.75	0.93	483.4–851.1	35	35.11	0.93	2.7
Glucose (g/L)	67.5–128.2	1043	5	3.16	0.96	68.9–122.6	261	3.39	0.97	3.5
Fructose (g/L)	74.7–128.8	1032	4	3.11	0.96	75.4–128.0	258	3.88	0.96	2.9
Ascorbic Acid (mg/100 g)	12.1–110.7	1040	6	6.99	0.94	16.7–105.6	260	7.32	0.93	2.7
Citric Acid (g/100 g)	5.9–11.2	1031	5	0.27	0.96	6.3–10.5	258	0.27	0.96	3.4

^a^ Number of samples used in calibration models. ^b^ Standard error of cross-validation. ^c^ Correlation coefficient of cross-validation. ^d^ Number of samples used in external validation models. ^e^ Standard error of prediction. ^f^ Correlation coefficient of prediction for validation. ^g^ Residual predictive deviation. Standard error of cross-validation (SECV) and standard error of prediction (SEP) are in units of the predicted parameters.
